# Dental maturity in children with celiac disease: a case–control study

**DOI:** 10.1186/s12903-020-01316-y

**Published:** 2020-11-09

**Authors:** Najlaa M. Alamoudi, Farah A. Alsadat, Azza A. El-Housseiny, Osama M. Felemban, Amani A. Al Tuwirqi, Rana H. Mosli, Omar I. Saadah

**Affiliations:** 1 Pediatric Dentistry Department, Faculty of Dentistry, King Abdulaziz University Hospital, Jeddah, Saudi Arabia; 2 Pediatric Dentistry Department, Faculty of Dentistry, Alexandria University, Alexandria, Egypt; 3 Clinical Nutrition Department, Faculty of Applied Medical Sciences, King Abdulaziz University, Jeddah, Saudi Arabia; 4 Pediatric Gastroenterology Unit, Department of Pediatrics, Faculty of Medicine, King Abdulaziz University, P.O Box 80205, Jeddah, 21589 Saudi Arabia

**Keywords:** Celiac disease, Children, Dental maturity, Dental age, Saudi arabia

## Abstract

**Background:**

Celiac disease (CD) is an immune-related enteropathy triggered by gluten ingestion in susceptible individuals. Oral manifestations of CD have been frequently described, although reports on dental maturity (DM) are scant. Thus, the aim of this study is to assess the prevalence of DM in CD patients and to test for possible predictors.

**Methods:**

This is a case–control study of children with CD and healthy controls between 2017 and 2020. A panoramic radiograph and comprehensive oral examination were performed for each participant. Dental age (DA) was measured according to Demirjian’s method and DM was calculated by subtracting the chronological age (CA) from the DA. Statistical analysis was performed to compare the DM between CD patients and controls, and a multivariate analysis was utilized to look for predictors of DM.

**Results:**

Two-hundred and eight participants (104 children with CD, and 104 healthy controls) were incorporated. The mean age for CD patients was 10.67 ± 2.40 years, and 10.69 ± 2.37 years for healthy controls (*P* = 0.971). CD patients had a higher prevalence of delayed DM than controls (62.5% vs. 3%, respectively). They also had a greater delay in DM than controls (− 7.94 ± 10.94 vs. 6.99 ± 8.77, *P* < 0.001). A multivariate analysis identified age between 6 and 7 years (β ± SE = 16.21 ± 2.58, *P* < 0.001), as the only predictor for DM.

**Conclusions:**

CD patients had a greater prevalence of delayed DM than controls. No predictors for DM could be found, except young age.

## Background

Celiac disease (CD) is an immune-mediated enteropathy targeting the mucosa of the small intestine and triggered by gluten ingestion in gentically susceptible individuals. [1]. CD may present with intestinal or extraintestinal symptoms, or without any symptoms at all (silent) [2]. Oral manifestations of CD have been reported, including apthous ulceration, delayed dental eruption (DE), and dental enamel defects [3].

Dental age (DA) determination is useful for forensic and legal purposes; for example, it can be used for determining the age of an individual with an unknown or unrecorded chronological age (CA). It can also help in the treatment of orthodontic cases. DA could be estimated by either the number of erupted teeth within the oral cavity, or the degree of tooth calcification assessed by radiography [4]. Dental eruption is affected by child’s socioeconomic status or individual characteristics, such as weight, height, gender, or culture [4, 5], although DE has been related to the general metabolism and growth of the body. Malnutrition has been reported in association with delayed DE [6]; children with a poor nutritional status may present with delayed DE. By contrast, children with a good nutritional status can have an advanced stage of DE [7]. Thus, delayed DA can be used as a clue for clinical diagnosis of patients with silent CD, in association with delayed growth and development [8]. A pediatric dentist should be aware of the various clinical features of CD in children for early diagnosis, since early intervention contributes to improved quality of life.

Several studies that examine the association between CD and DA have been published [9–12]. These show that CD patients tend to have delayed DE in comparison with healthy controls, attributable to the general delay in growth and development found in CD patients [8]. Some authors relate delayed DE to the effect of poor nutritional status often observed in CD patients [9].

Dental maturity (DM) reflects the difference between the dental age (DA) and chronological age (CA). Its determination provides more information on the eruption of teeth and dental development, focusing on the developmental process rather than local or environmental factors [6], enabling, for example, the development of an orthodontic treatment plan [10]. However, to the best of our knowledge, there is a limited number of published studies on children with CD related to their DM. Thus, we aim to examine the DM in children with CD and to compare this with healthy controls to look for possible predictors of DM.

## Methods

### Study population

This is a case–control study based on “The Reporting of Observational Studies in Epidemiology (STROBE)” guidelines for reporting case control studies [11]. The sample size was calculated by applying an equation for calculating sample size in case–control studies [12], using 80% power and an alpha level of 0.05. A total number of 190 participants was the minimum requirement (95 for the CD group and 95 for the control group) [13].

The inclusion criteria were the following: children with a CA of 6–14 years with biopsy-proven CD diagnosed according to criteria set out by the European Society of Pediatric Gastroenterology Hepatology and Nutrition (ESPGHAN) [14]. For the control group, only healthy children with ASA 1 status (approved on October 15, 2014 by the American Society of Anesthesiologists) were included. The exclusion criteria were the following: children with mental or physical disabilities or congenital abnormalities and those not having bilateral missing premolars, since DA calculation would not be possible by the traditional method in such cases [15].

CD children were recruited from the Pediatric Celiac Disease Clinic in King Abdulaziz University Hospital (KAUH) in the period between September 2017 and February 2020. Simultaneously, healthy controls were obtained by giving each participant 5 letters of invitation to participate in the study to distribute to their classmates. Parents that replied and agreed to participate were requested to bring their child to King Abdulaziz University Dental Hospital (KAUDH) to be included in the study; this process was iterated until the desired number of healthy controls was obtained.

Ethical considerations in accordance with the Declaration of Helsinki were followed throughout this study. This study was approved by the Research Ethics Committee of the Faculty of Dentistry in King Abdulaziz University (KAU) (Ref: 078-09-17). Parents had to sign an informed parental consent form to give permission for the participation of their child.

### Data collection

A panoramic radiograph was taken in the radiology department. Following this, the parents of the participating children, along with their child, were taken to the dental clinic to complete a personal interview about the child, regarding demographic data, number and sequence of child sibling(s), child dietary habits, and socioeconomic status—specifically parental education and family income. Parental education was categorized as ≤ 12 years or > 12 years of education. Family income was categorized into low [less than 5000 Saudi Arabian Riyals (SAR)], middle (from 5000 to 10,000 SAR), and high (more than 10,000 SAR). Subsequent to the interview, a comprehensive oral clinical examination and dental prophylaxis were carried out.

On a separate day, a non-investigator coded the radiographs, in order for the single trained pediatric dental examiner to be blinded during radiographic DA calculation, to avoid bias. The DA was then measured using the panoramic radiograph, following the Demirjian method [15].

The dietary habits were recorded by interviewing both child and parent, using a checklist. This list corresponds to the frequency intake of the five food groups, as set out by the WHO [16]. For each food group, a rating on dietary intake was recorded on a scale of 1–4, where 1 corresponds to several times a day, 2 is daily, 3 is sometimes, and 4 never. Dietary intake was dichotomized, where ratings of 1 and 2 indicated *yes*, and ratings of 3 and 4 indicated *no*.

CA represents the time elapsed since birth, and DA estimates a person’s age based on the magnitude of teeth formation in the jaws [4]. For each participant, the CA was calculated from the date of examination according to his or her date of birth. The DA was calculated by the assessment of the left permanent mandibular teeth in the following order: 2nd molar, 1st molar, 2nd premolar, 1st premolar, canine, lateral incisor, and central incisor. Teeth were rated based on the “A to H” scale, following the given criteria and diagrams at every stage. Each tooth was then given a numerical score that was extracted from predetermined tables for the respective gender. The sum of the seven numerical scores yielded a maturity score, which was subsequently interpreted on the given tables to determine the corresponding DA. In case of a single missing premolar on the left side, the corresponding premolar on the right side was used instead [17]. Participants with bilateral loss of premolars were excluded. The DA was then compared to the CA to assess the state of dental growth and development.

The mean difference between DA and CA was calculated (DM = DA − CA) to assess DM. If the difference was positive, it corresponds with advanced DM, while if it was negative it corresponds with delayed DM [4]. To calculate the difference in months, the mean difference was multiplied by 12. The mean differences were then arranged into three categories: mild (up to 12 months), indicating values within the normal age range, moderate (from 12 to 24 months), and severe (more than 24 months); the latter two categories indicating values outside the normal age range [4, 18].

### Inter- and intra-rater reliability assessment

Inter-rater reliability for the DA calculation was tested using Demirjian’s method by the principal investigator through examining ten panoramic radiographs, under the supervision of a trained pediatric dentist; the process was then repeated two weeks later to calculate intra-rater reliability. For a DA calculation using Demirjian’s method, inter- (with the trainer pediatric dentist) and intra-rater reliability was calculated using the Statistical Package for the Social Sciences (SPSS), version 21 (SPSS, Inc., Chicago, IL, USA)..

### Statistical analysis

Descriptive statistics were used to examine sample characteristics: continuous variables were summerized as means and standard deviations, while counts and percentages were used for categorical variables. The association between the covariates (i.e., personal characteristics and socio-demographic variables, such as family income, parental education, and dietary factors) and the study outcome (DM) were initially evaluated by stratifying the subjects into a CD group and control group. Associations between the covariates and the study outcomes were then evaluated using chi-squared tests, Fisher’s exact tests, independent *t* tests, or analysis of variance (ANOVA) tests. Post-hoc analysis using Tukey correction was used in cases of significant results in ANOVA tests. After that, mutivariate analyses were carried out (mutiple linear regression models) to predict the DM, to check for interaction, and to control for possible confounding between CD and other covariates. A multiple linear regression analysis was also used to explore possible predictors of DM in CD patients. A *P *value of < 0.05 was set as statistically significant. The Statistical Package for the Social Sciences (SPSS), version 21 (SPSS, Inc., Chicago, IL, USA) was used for the statistical analysis.

## Results

### Sample charactristics

Two-hundred and eight participants (104 children with CD, and 104 healthy controls) were included in the analysis. A total of 149 CD patients were contacted, and 520 letters of invitation were distributed to the classmates of CD children. The response rate of the CD group was 69.80%, while the response rate of the control group was 20%. In both the CD and control groups, 50% of the participants were girls. No statistically significant differences were found between the socioeconomic distribution of the two groups with respect to family income (*P* = 0.874), mother education (*P* = 0.052), and father education (*P* = 0.781). Children in the CD group were less likely to report daily intake of protein (76.0%), compared to the control group (80.8%) and the difference was statistically signifcant (*P* = 0.027). Thirteen of the children with CD were taking growth hormones (GHs) (Table 1).

**Table 1:** Characteristics of the study population

Variables	Celiac N = 104 n (%)	Control N = 104 n (%)	*P* value^‡^
(A) Sociodemographic data			
Gender			
Girls	52 (50.0)	52 (50.0)	1.000
Boys	52 (50.0)	52 (50.0)	
Age categories (years)			
6–7	17 (16.3)	16 (15.4)	0.980
8–10	33 (31.7)	33 (31.7)	
11–14	54 (51.9)	55 (52.9)	
Child sibling sequence			
The first	22 (21.2)	27 (26.0)	0.599
In the middle	62 (59.6)	55 (52.9)	
The last	20 (19.2)	22 (21.2)	
Family income			
Low	32 (30.8)	35 (33.7)	0.874
Middle	35 (33.7)	32 (30.8)	
High	37 (35.6)	37 (35.6)	
Mother education			
≤ 12 years	62 (59.6)	48 (46.2)	0.052
> 12 years	42 (40.4)	56 (53.8)	
Father education			
≤ 12 years	58 (55.8)	56 (53.8)	0.781
> 12 years	46 (44.2)	48 (46.2)	
(B) Dietary details			
Grains daily intake			
Yes	79 (76.0)	85 (81.7)	0.308
No	25 (24.0)	19 (18.3)	
Protein daily intake			
Yes	70 (67.3)	84 (80.8)	0.027*
No	34 (32.7)	20 (19.2)	
Dairy daily intake			
Yes	51 (49.0)	53 (51.0)	0.782
No	53 (51.0)	51 (49.0)	
Vegetable daily intake			
Yes	34 (32.7)	30 (28.8)	0.548
No	70(67.3)	74 (71.2)	
Fruit daily intake			
Yes	40 (38.5)	52 (50.0)	0.094
No	64 (61.5)	52 (50.0)	
(C) Growth hormone			
Yes	13 (12.5)	NA	
No	91 (87.5)		

*N *the total number of participants, *n *number of participants in each group, *CI* confidence interval

^**‡**^Chi-square test/Fisher exact test

**Fig. 1:**
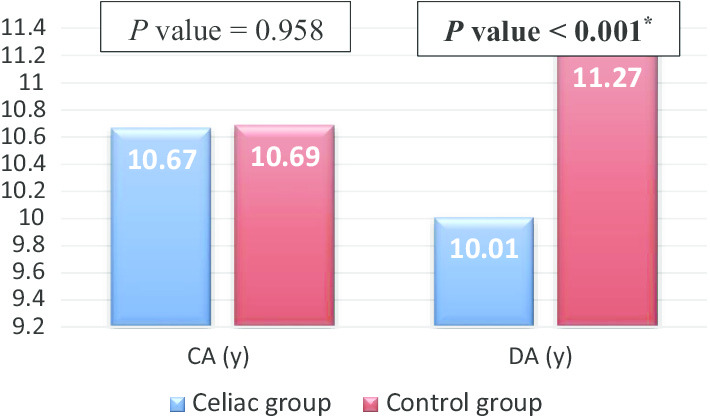
Comparison between chronological age (CA), and dental age (DA) in the study and control groups

**Fig. 2:**
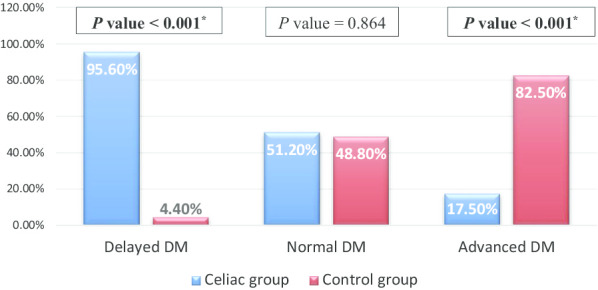
Comparison in the degree of dental maturity (DM) between the study and control groups

The mean CA was 10.67 ± 2.40 (95% CI 10.21–11.14) years in the children with CD, and 10.69 ± 2.37 (95% CI 10.23–11.15) years in the healthy controls (*P* = 0.958). Children with CD had significantly lower mean DA compared to controls (10.01 years ± 2.05; 95% CI 9.62–10.41 vs. 11.27 years ± 2.42; 95% CI 10.81–11.75, *P* < 0.001) (Fig. 1). Therefore, children with CD had a delayed DM of 0.66 ± 0.91 years, corresponding to 7.94 ± 10.94 months, while the healthy controls had an advanced DM of 0.58 ± 0.73 years, corresponding to 6.99 ± 8.77 months (*P* < 0.001). The DM categories among all participants showed that 95.60% were CD children in the category of delayed DM, (*P* < 0.001). In the category of advanced DM, 82.50% were healthy controls (*P* < 0.001) (Fig. 2).

In the CD group, 65 patients (62.5%) had delayed DM, 22 patients (21.2%) had normal DM, and 17 patients (16.3%) had advanced maturity. In the control group, only 3 participants (2.9%) had delayed DM, 21 participants (20.2%) had normal DM, and 80 participants (76.9%) had advanced DM.

### DM categories in children with CD and healthy controls

**Fig. 3:**
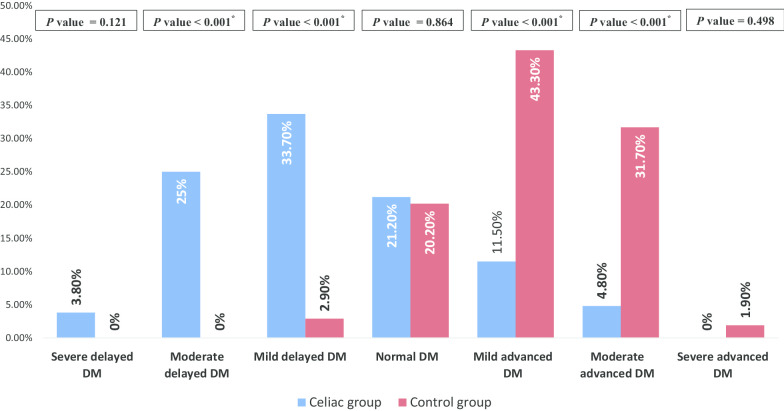
Percentage distribution of dental maturity (DM) categories among the whole sample

Grading the DM for the CD group, 22 patients (21.2%) had normal maturity, 35 patients (33.7%) had mild delay, 26 patients (25%) had moderate delay, 4 patients (3.8%) had severe delay, 12 patients (11.5%) had mild advanced, only 5 patients (4.8%) had moderate advanced, and none had severe advanced. Grading the DM for the healthy controls showed that 21 participants (20.2%) had normal maturity, 45 participants (43.3%) had mildly advanced, 31 participants (29.8%) had moderately advanced, 4 participants (3.8%) had severly advanced, 3 participants had mild delay, and none had moderate or severe delay (Fig. 3).

### Bivariate analysis of the mean difference between DA and CA in children with CD vs. healthy controls.

A bivariate analysis of the DM in months (difference between DA and CA) by demographic, SES, and dietary variables was carried out speparately for CD and control groups. Boys in the control group had signifcantly higher mean DM of 8.77 ± 9.27 months (95% CI 6.19–11.35), compared to girls (5.22 ± 7.94 months; 95% CI 3.01–7.43) (*P* = 0.034). Moreover, the youngest age category (6–7 years) in general had higher DM of 5.65 ± 7.66 months (95% CI 1.71–9.59), corresponding to an advanced DM, which was significantly different from the other two age groups (*P* < 0.001); the middle age group (8–10 years) had a mean DM of − 8.52 ± 07.50 months (95% CI − 11.18 to − 5.86), corresponding to a delayed DM; and the oldest age group (11–14 years) had a mean DM of -11.85 ± 10.28 months (95% CI − 14.66 to − 9.05), corresponding to a delayed DM.

Family income was found to be associated with the DM in the CD group (*P* = 0.016). The middle-income group (between 5000 and 10,000 SAR) had a mean DM of − 11.32 ± 9.70 months (95% CI − 14.66 to − 7.99), corresponding to a delayed DM, which was significantly different from the highest-income group (> 10,000 SAR), which had a mean DM of − 4.07 ± 10.90 months (95% CI − 7.70 to − 0.43), corresponding to a delayed DM. Mother education was also associated with DM in the CD group (*P* = 0.042). Children whose mothers had less than 12 years of education had a mean DM of − 9.24 ± 10.16 months (95% CI − 11.91 to − 6.57), corresponding to delayed DM, which was significantly different from those whose mothers had more than 12 years of education, with a mean DM of − 6.29 ± 11.75 months (95% CI − 9.78 to − 2.80), corresponding to a delayed DM.

In the control group, daily intake of vegetables was significantly associated with DM (*P* = 0.048). The mean CA and DA and the mean DM did not show a significant difference between children with CD that were taking growth hormones (GHs) and those that were not taking them (*P* = 0.783). The mean DM of children taking GH was 5.64 ± 9.36 months (95% CI 0.036–11.4), while the mean DM of those that were not taking GHs was 6.48 ± 10.44 months (95% CI 4.32–8.76) (Table 2).

**Table 2:** Bivariate analysis of different covariates and DM in months stratified by CD status

Variables	Celiac (n = 104)			Controls (n = 104)		
	DM Mean ± SD	95% CI	*P *value^†^	DM Mean ± SD	95% CI	*P *value^†^
Gender						
Boys	− 9.85 ± 8.99	− 12.35 to − 7.35	0.074	8.77 ± 9.27	6.19–11.35	0.038*
Girls	− 6.02 ± 12.38	− 9.47 to − 2.57		5.22 ± 7.94	3.01–7.43	
Age categories (years)						
6–7	5.65^a^ ± 7.66	1.71 to 9.59	< 0.001*	8.40 ± 4.57	5.97–10.83	0.745
8–10	− 8.52^b^ ± 7.50	− 11.18 to − 5.86		6.33 ± 9.63	2.92–9.75	
11–14	− 11.85^b^ ± 10.28	− 14.66 to − 9.05		6.98 ± 9.23	4.49–9.48	
Child siblings sequence						
The first	− 9.71 ± 10.31	− 14.28 to − 5.15	0.261	6.32 ± 9.73	2.47–10.17	0.769
In the middle	− 8.42 ± 11.22	− 11.27 to − 5.57		7.59 ± 8.74	5.23–9.95	
The last	− 4.48 ± 10.48	− 9.38 to 0.43		6.34 ± 7.87	2.84–9.83	
Family income						
Low	− 8.70^ab^ ± 11.17	− 12.73 to − 4.67	0.016*	5.32 ± 8.90	2.26–8.38	0.171
Middle	− 11.32^a^ ± 9.70	− 14.66 to − 7.99		9.29 ± 5.87	7.17–11.40	
High	− 4.07^b^ ± 10.90	− 7.70 to − 0.43		6.59 ± 10.40	3.13–10.06	
Mother education						
≤ 12 years	− 9.73 ± 10.08	− 12.29 to − 7.17	0.042*	5.78 ± 8.02	3.46–8.12	0.196
> 12 years	− 5.29 ± 11.72	− 8.94 to − 1.64		8.03 ± 9.31	5.53–10.52	
Father education						
≤ 12 years	− 9.24 ± 10.16	− 11.91 to − 6.57	0.174	**5.84** ± 8.47	3.57–8.11	0.149
> 12 years	− 6.29 ± 11.75	− 9.78 to − 2.80		8.34 ± 9.01	5.72–10.95	
Grains daily intake						
Yes	− 7.71 ± 10.77	− 10.13 to − 5.30	0.717	7.45 ± 8.66	5.58–9.32	0.265
No	− 8.63 ± 11.64	− 13.44 to − 3.82		4.96 ± 9.20	0.53–9.39	
Protein daily intake						
Yes	− 7.61 ± 10.33	− 10.07 to − 5.15	0.666	6.91 ± 9.03	4.95–8.87	0.841
No	− 8.60 ± 12.23	− 12.87 to − 4.34		7.35 ± 7.79	3.71–10.99	
Dairy daily intake						
Yes	− 7.90 ± 9.95	− 10.69 to − 5.10	0.974	7.80 ± 8.74	5.39–10.21	0.342
No	− 7.97 ± 11.91	− 11.25 to − 4.69		6.16 ± 8.81	6.68–8.63	
Vegetable daily intake						
Yes	− 6.94 ± 9.48	− 10.25 to − 3.63	0.520	9.66 ± 10.25	5.83–13.479	0.048*
No	− 8.42 ± 11.62	− 11.19 to − 5.65		5.94 ± 7.92	4.08–7.75	
Fruit daily intake						
Yes	− 5.58 ± 11.13	− 9.14 to − 2.02	0.082	8.05 ± 8.76	5.61–10.49	0.223
No	− 9.41 ± 10.64	− 12.07 to − 6.75		5.94 ± 8.74	3.61–8.38	
Growth hormone						
Yes	5.64 ± 9.36	0.04–11.4	0.783	NA		
No	6.48 ± 10.44	4.32–8.76				

Bold values indicate significant level of *P* values

Means sharing the same superscript are not significantly different from each other (Tukey’s post hoc test, *P* value ≥ 0.05). Means that have different alphabetical letter superscript are significantly different from each other (Tukey’s post hoc test, *P* value < 0.05)

*N *total number of participants, *n *number of participants in each group, *CI* confidence interval, *CA* chronological age, *DA* dental age, *DM* dental maturity, *CD* celiac disease

*P value < 0.05

^†^Independent *t* test or ANOVA

### Predictors of DM in the CD and control groups

A multiple linear regression model was applied to confirm the relationship between CD and DM, while controlling for confounding variables. Children with CD had a signifcanlty delayed DM by about 15 months on average (β ± SE, − 14.84 ± 1.31; 95% CI − 17.41 to − 12.26, *P* < 0.001) compared to control subjects, after controlling for other variables. Children aged 6–7 years had advanced DM by 9.32 ± 1.88 months (95% CI 5.61–13.03, *P* < 0.001) in comparison with the 11–14 year-old children after controlling for other variables (Table 3).

**Table 3:** Multiple linear regression analysis for dental maturity in months among the whole sample

Variable	Category	Β estimate	SE	95% CI	*P* value
Celiac disease	Yes	− 14.84	1.31	− 17.41 to − 12.26	< 0.001*
	No	Ref			
Gender	Girls	− 0.71	1.32	− 3.31 to 1.90	0.594
	Boys	Ref			
Age (years)	6–7	9.32	1.88	5.61 to 13.03	< 0.001*
	8–10	0.93	1.47	− 1.96 to 3.82	0.526
	11–14	Ref			
Family Income	Low	− 1.17	1.72	− 4.56 to 2.23	0.499
	Middle	− 1.71	1.60	− 4.88 to 1.45	0.287
	High	Ref			
Mother education	≤ 12 years	− 1.99	1.45	− 4.85 to 0.86	0.169
	> 12 years	Ref			
Vegetable daily intake	Yes	2.65	1.41	− 0.13 to 5.41	0.062
	No	Ref			

Bold values indicate significant level of *P* values

*SE* standard error, *CI* confidence interval

*P value < 0.05

### Predictors of DM in CD group

A multiple linear regression model was used to explore possible predictors of DM in the CD group (n = 104). Young age was found to be the only significant predictor for DM. Children in the youngest age group (6–7 years) had significantly advanced DM by 16.21 ± 2.58 months (95% CI 11.09–21.32, *P* < 0.001) compared to 11–14 year-old children (Table 4).

**Table 4:** Multipe linear regression to determine predictors of DM in months in CD group

Variable	Category	Β estimate	SE	95% CI	*P *value
Age (years)	6–7	16.21	2.58	11.09 to 21.32	< 0.001*
	8–10	3.38	2.01	− 0.61 to 7.37	0.096
	11–14	Ref			
Family Income	Low	− 1.46	2.42	− 6.26 to 3.35	0.549
	Middle	− 4.30	2.22	− 8.70 to 0.11	0.056
	High	Ref			
Mother education	≤ 12 years	− 1.64	2.02	− 5.65 to 2.37	0.418
	> 12 years	Ref			

Bold values indicate significant level of *P* values

*DM* dental maturity, *SE* standard error, *CI* confidence interval

*P value < 0.05

### Inter and intra-rater reliability assessment

Intra-class correlation coefficient (ICC) for inter-rater agreement was carried out for each tooth separately on the lower left quadrant; the score ranged from 0.96 to 1, which indicated excellent reliability [19]. Also, intra-rater reliability was performed for each tooth separately; the score ranged from 0.95 to 1, which likewise indicated excellent reliability [19].

## Discussion

The purpose of this study was to assess dental maturity (DM) in children with CD. The presence of delayed DM in children is an important consideration and of acute interest to pediatric dentists. It is regarded as a key clinical factor that should be examined to identify children with CD, separate to the presence of gastrointestinal symptoms, informing decision-making in suspected cases. Early recognition and diagnosis help in enabling prompt implementation of a gluten-free diet (GFD), which results in better treatment and militates against complications [20, 21].

Dental age (DA) can be assessed clinically—through the enumeration of erupted teeth, as well as radiographically, which has been the approach of the present study. The reported advantage of the clinical method was primarily economic, excluding the need for costly additional equipment. However, this may be considered a drawback, since dental eruption (DE) dates have a wide range that can depend on various environmental or dental factors, such as the existing space within the dental arches, early extraction of primary teeth, or periapical abscesses causing rarifaction of bone. By contrast, the reported advantages of the radiographic method are that it depends exclusively on the development of teeth, regardless of local or enviromental factors [6]. Accordingly, the Demirjian method is the recommended method for assessing DM in children in Saudi Arabia [10].

Multiple studies reported DA in CD using the clinical assessment of DE [9, 22–24]. By contrast, the current study assessed DA/DM radiographically. We found delayed DM in 62.5% of children in the CD group and 3% of children in the healthy control group. These findings are broadly in line with a number of previously published case–control studies [9, 20–24], where a range of 20–70% for children with CD vs. 7–20% for healthy controls were reported. The prevalence of delayed DA was similar in the present study to the findings of these two studies, using a similar methodology of assessing DA radiographically [8, 9], with a range from 56.7 to 70% in children with CD. However, the prevalence of delayed DM in our study varied from those studies in that DA was assessed clinically, by counting the number of erupted teeth [9, 22–24], with a range from 20 to 38% in children with CD. Greater prevalence of delayed DE has likewise been reported in CD patients than controls [8], and was considered a significant oral manifestation of CD in the appraisals of several studies [23, 25, 26].

The delayed DA in CD might be related to malnutrition and malabsorption of nutrients or vitamins necessary for dental development, and retarded growth, as reported in a number of studies [8, 9, 20, 22, 27]. This analysis is also supported by one study which showed that the adequate intake of carbohydrates and fruits in children helped to militate against delayed DE [28].

The present study found an average delayed DM of about 8 months in the CD group and an average advanced DM of 7 months in the control group. This difference in DM in CD patients was broadly in line with that of an Italian study (6–7 months), although in that study there was a delay of only 1 month in the healthy controls [21]. However, variation of ± 6 months was considered a normal finding in another study [22]. With regard to delayed DE, 6 months delay was considred within a normal range in a study that found delayed DE of 1.4 years in CD children, and delay of less than one year in the control group [24]. However, another study considered delayed DE after eight months beyond the normal eruption time [23]. In our study, we calculated the mean difference between CA and DA to assess for dental maturity, as defined previously [4, 18]. A difference of approximately 3 months was considered normal, and delayed or advanced dental maturity is within the standard range of 12 months, as broadly defined in the literature [18]. Some investigators, however, have considered a delay of one month to be a minimal delay [20]. These differences in definitions of delayed DA or DE might have led to a considerable variety of conclusions regarding CD.

To the best of our knowledge, a limited number of studies have evaluated dental maturity based on DA in children with CD [23, 29, 30]. Studies [23, 29] are relatively old, [28] is not written in English, and [29] also contains few details regarding dental maturity. However, the study design used in these publications, in which a CD group and control group of healthy children are examined concurrently, helped in the present study in documenting abnormalities related to a CD group in comparison to a control group. Indeed, we found that children with CD had a greater likelihood of delayed DM in comparison with healthy controls. One key study, however, reported variation in DA among children with CD on a GFD, which could explain the effect of a GFD in reversing dental manifestations of CD [20]. Our healthy controls, by contrast, showed more advanced DM, in agreement with a study that reported advanced DA in a healthy population [31].

A bivariate analysis was performed to determine factors which could influence DM. The effect of gender on tooth development has yielded inconsistent results in the literature. In the present study, boys in the control group had a more advanced DM, which constrast with the findings of some previous studies in normal children and in children with a cleft lip and palate [10, 27, 32, 33]. Perhaps the difference in DM between genders is also related to genetic, hormonal, and envionmental factors [5, 27]. Furthermore, a study from Italy has found no gender difference between children with delayed DA [20]. In the present study, the older age group in children with CD was associated with greater delayed DM, in contrast to the younger age group, similar to the findings of the aforementioned study of patients with a cleft lip and palate [33].

Higher family income and higher maternal education were found to be a preventive factors for delayed DM, which may be a product of a presumably richer nutritional supply and a higher standard of dental care. Lower socioeconomic status, and particularly poverty, where families cannot afford appropriate and nutritious food, may affect the provision of a GFD; this was reported in relation to DE in one study [28]. Daily vegetable consumption in healthy controls was found to be a protective factor against delayed tooth maturity. Perhaps this was because controls may not have problems in their absorption of nutrients, specifically vitamin and minerals, present in vegetables. This was supported by a study that illustrated that the intake of vegeables helps in maturation of the masticatory apparatus, including teeth, used for chewing food [28].

The present study may have some moderate limitations. Regular consumption of a GFD has been shown to enhance growth of teeth in the jawbones [20], and found to have a constructive effect on increasing DM [21]. In the present study, we were not able to assess compliance with a GFD. Such data would have been better taken in a longitudinal study design, which we recommended to be carried out in future work. Indeed, this study has a cross-sectional nature, which makes us unable to determine a cause–effect relationship between variables. Another limitation of the food checklist is that this method is not representative of the exact amount of daily food intake [34]. Also, CD patients that were taking GHs were not excluded, since 19.7% of patients with CD had been found with a GH deficiency, requiring GH treatment, despite being on a GFD [35]. The main strength of this study is that it is perhaps the only recent study to determine effectively the significance of DM in children with CD.

## Conclusions

This case–control study illustrates that children with CD have a generally greater delay of DA and DM than healthy controls. The prevalence of delayed DM in children with CD is 62.5%. Young age, mother education, and family income are significant factors associated with delayed DM. No predictors could be detected with DM in children with CD except young age. Therefore, more longitudinal studies are needed to find more predicors for DM in children with CD.

## Data Availability

The datasets used and/or analysed during the current study available from the corresponding author on reasonable request.

## Abbreviations

ANOVA: Analysis of variance
ASA: American Society of Anesthesiologists
CA: Chronological age
CD: Celiac disease
CI: Confidence interval
DA: Dental age
DE: Dental eruption
DM: Dental maturity
ESPGHAN: European Society of Pediatric Gastroenterology Hepatology and Nutrition
GFD: Gluten-free diet
GH: Growth hormone
KAU: King Abdulaziz University
KAUDH: King Abdulaziz University Dental Hospital
KAUH: King Abdulaziz University Hospital
SAR: Saudi Arabian Riyals
SPSS: Statistical Package for the Social Sciences
WHO: World health Organization
